# Frail older adults’ challenges in using telehealth services: A scoping review

**DOI:** 10.1177/20552076261479799

**Published:** 2026-08-20

**Authors:** Kristina Åhlund, Ann Svensson, Lilisbeth Perestelo-Perez, Ángel Acebes, Johannes Hobbelen, Margareta Karlsson

**Affiliations:** 1 Department of Health Care Sciences, 42749 University West, Trollhättan, Sweden; 2 Department of Research and Development, Region Västra Götaland, NU-Hospital Group, Trollhättan, Sweden; 3 Department of Orthopedics, Institute of Clinical Science, 70712 University of Gothenburg Sahlgrenska Academy, Gothenburg, Sweden; 4 School of Business, Economics and IT, 42749 University West, Trollhättan, Sweden; 5 Department of Quality and Health Technology, 56627 University of Stavanger, Stavanger, Norway; 6 Department of Industrial Management, Industrial Design and Mechanical Engineering, Faculty of Engineering and Sustainable Development, 3485 University of Gävle, Gävle, Sweden; 7 Evaluation Unit, Canary Islands Health Research Institute (IISC), RICAPPS, RedETS, 375043 Canary Islands Health Service (SESCS), Tenerife, Spain; 8 Institute of Biomedical Technologies, Department of Basic Medical Sciences, 16749 University of La Laguna, Tenerife, Spain; 9 Research Group Healthy Ageing, Allied Health Care and Nursing, 3645 Hanze University of Applied Sciences, Groningen, Netherlands; 10 University Medical Center, Department of General Practice and Elderly Care Medicine, 3647 University of Groningen, Groningen, Netherlands

**Keywords:** challenges, digital literacy, frailty, older adults, telehealth

## Abstract

**Objective:**

Telehealth is increasingly used to expand access to healthcare, but rapid digitalization risks exacerbating exclusion among frail older adults, who often face intersecting limitations related to health, resources, and digital competence. As this group frequently has complex care needs, mapping their experiences is essential to ensure equitable access to digital care. The aim was to map the challenges frail older adults experience when using telehealth services.

**Methods:**

This scoping review followed Arksey and O’Malley’s framework and PRISMA-ScR guidelines. PubMed, CINAHL, Scopus, and Web of Science were searched for peer-reviewed empirical studies published in English (January 2015–June 2026). Studies were included if they addressed relevant challenges from the perspectives of frail older adults or those with multimorbidity, disability, cognitive impairment, or dependency. Data were charted and analyzed narratively.

**Results:**

Of 7200 studies identified, 22 were included. All of them used qualitative methods and were conducted in Europe, North America, and Asia; only four applied formal frailty assessments. Five domains were identified: 1) digital accessibility, 2) digital skills, 3) health status, 4) digital support, and 5) financial resources.

**Conclusions:**

As frail older adults face multiple, interconnected challenges to telehealth use, including technological and environmental constraints, limited digital competence, health-related impairments, insufficient support, and financial challenges, services should be designed to align with their needs and life circumstances. Accessible design, structured support, affordable connectivity, and active involvement of frail older adults are essential to ensure equitable and inclusive digital healthcare.

## Introduction

Across Europe, the proportion of individuals living into older age is increasing.^1^ Aging occurs along a continuum ranging from robustness to frailty, resulting in substantial variation in physical, cognitive, and social functioning among individuals of the same chronological age.^2^ Frailty describes the complex interplay among aging, disability, and multimorbidity, and reflects reduced resilience to stressors.^3^ It is associated with poorer medical outcomes, including functional decline, dependency, increased healthcare utilization, and mortality.^3,4^

To sustain effective healthcare delivery amid profound demographic changes, many countries now face considerable strain on their healthcare resources. In response, healthcare systems are rapidly implementing digital solutions, as digitalization is widely regarded as a key strategy for strengthening system capacity and addressing increasing workloads. Globally, digital health initiatives are intended to improve health by accelerating the adoption of appropriate and scalable digital technologies.^5^

Telehealth involves the use of electronic and telecommunication technologies to provide healthcare services at a distance, allowing interactions between patients and clinicians without physical presence.^6^ It encompasses a range of care modalities, including asynchronous services (e.g., communication via secure patient portals), synchronous interactions (e.g., real-time video consultations), and remote patient monitoring (e.g., digital tracking of health data over time), and has the potential to enhance system efficiency through the more effective use of limited resources.^7^ It has also reshaped the role of healthcare professionals, shifting it toward more consultative and supportive functions that facilitate shared decision-making and greater patient participation in their own care.^8^ For patients, telehealth can improve access to services, enable more autonomous self-management, and support personalized treatment pathways, thereby promoting more equitable care regardless of geographic location. However, the rapid expansion of telehealth solutions also transfers greater responsibility to individuals. Those without adequate access to technology or sufficient digital competence risk marginalization, and a pronounced digital divide is already evident among older adults.^9^

Equity in healthcare entails ensuring that all individuals can achieve their full health potential.^10^ To support health management, disease prevention, and engagement in health-promoting behaviors, both digital literacy and health literacy are essential.

Health literacy encompasses not only the comprehension of health information but also the ability to make informed decisions based on that information.^11^ Sociodemographic, economic, and health-related factors, such as chronic conditions, frailty, and disabilities, have been identified as important determinants associated with poor health literacy.^12^ Even when it comes to digital literacy, similar challenges persist, with older adults often experiencing greater difficulty navigating digital services.^8^ Although digital adoption increased markedly with the COVID-19 pandemic, older adults with more complex care needs continue to face substantial difficulties when engaging with telehealth services.^10,13^

Telehealth has the potential to contribute to more equitable care, and a recently published qualitative study^14^ suggests that it can be perceived as a flexible resource for self-management in patients with long-term conditions, promoting autonomy through symptom monitoring and enabling care to be accessed when needed. Convenient contact with healthcare professionals may also enhance patients’ sense of safety.^14^ However, these benefits rely on active engagement and adequate digital skills, and for those who lack such competencies, telehealth may instead act as a barrier and risk reinforcing existing healthcare inequities.

Consequently, frail older adults use digital tools less frequently than do their non-frail peers, even though they may have the most to gain from such technologies.^15^ This gap underscores the need for targeted support from healthcare professionals with sufficient digital skills, as well as clear and structured efforts to strengthen older adults’ digital health competence and confidence.^16^

As population ageing coincides with the continued expansion of telehealth services, healthcare systems must ensure that digitalization supports health and equity rather than reinforcing existing vulnerabilities. Developing inclusive and individually adapted telehealth services requires a clearer understanding of the challenges experienced by frail older adults themselves. However, current knowledge remains limited across different telehealth contexts. Therefore, this scoping review aims to map the challenges experienced by frail older adults when using telehealth services.

## Methods

To enhance methodological transparency and rigor, the review process followed the methodological framework of Arksey and O’Malley,^17^ comprising five major steps: 1) identifying the research question, 2) identifying relevant studies, 3) selecting the studies, 4) charting the data, and 5) collating, summarizing, and reporting the results. The reporting process adhered to the Preferred Reporting Items for Systematic Reviews and Meta-Analyses extension for Scoping Reviews (PRISMA-ScR)^18^ guidelines and the review was pre-registered with the Open Science Framework (OSF) under https://doi.org/10.17605/OSF.IO/DM8U7.

### Search strategy

The first step of Arksey and O'Malley’s framework,^17^ identifying the research question, was guided by the Population–Concept–Context (PCC) mnemonic,^19^ which informed the development of the research questions, eligibility criteria, and search strategy. Accordingly, the search strategy was structured around older adults with frailty (population), challenges and barriers related to the use of digital technologies (concept), and the use of digital health technologies (context). Synonyms and related terms for each PCC component were included to maximize the identification of relevant studies.

A systematic literature search was performed in four electronic databases, i.e., PubMed (including MEDLINE and PubMed Central), CINAHL, Scopus, and Web of Science, which are considered to encompass interdisciplinary research in healthcare. Consistent with the second step of Arksey and O'Malley’s framework,^17^ relevant studies were identified through systematic database searches. An initial search in PubMed was conducted to identify relevant terms and synonyms, followed by preliminary searches to refine the strategy. The final search string was then adapted for use across all databases. The structured search, including all identified keywords and index terms, was conducted in August 2025 and was subsequently updated with an additional search in June 2026, which yielded one additional study. For search details, see supplementary file.

The systematic search used the following Boolean search string: (Frail* OR multimorbidity OR comorbidity OR multi-disease* OR patient* complex needs OR senior* OR older adult* OR elderly) AND (Digital challenge* OR digital divide OR technology gap OR tech struggle* OR digital aversion OR digital barrier* OR digital competence OR digital tools accessibility OR digital exclusion) AND (digital* OR e-health OR m-health OR telehealth OR health informatics OR health information OR digital communication OR remote care OR telemedicine) AND (Experience* OR perception* OR perspective*). Quotation marks were not used to allow a broader search, enabling automatic term mapping and the inclusion of variations and related terms, which supports the exploratory aim of a scoping review.

### Eligibility criteria

The inclusion criteria were empirical scientific articles published in English from 2015 onwards, focusing on frail older patients’ perspectives on challenges with telehealth services. The included studies must have undergone scientific peer review before publication. To ensure that the focus remained on the experiences of older adults living with frailty, studies that did not include a frailty assessment were considered for inclusion based on participants’ characteristics. Multimorbidity, disability, need for assistance in daily life, and symptom burden were used as proxies for frailty, as these characteristics are closely associated with the condition and are commonly incorporated into frailty assessment instruments.^4^ For example, the Clinical Frailty Scale^20,21^ incorporates information on physical functioning, dependence in activities of daily living, symptom-related limitations in daily life, and where applicable, dementia severity when assessing overall frailty. Another widely used frailty approach is Fried’s Frailty Phenotype,^22^ which focuses on low physical activity, muscle weakness, exhaustion, weight loss, and slowness. Similar dimensions are reflected in shorter screening instruments such as FRESH,^23^ including mobility, fatigue, fear of falling, and dependency in daily activities.

### Selection process

**Figure 1.**
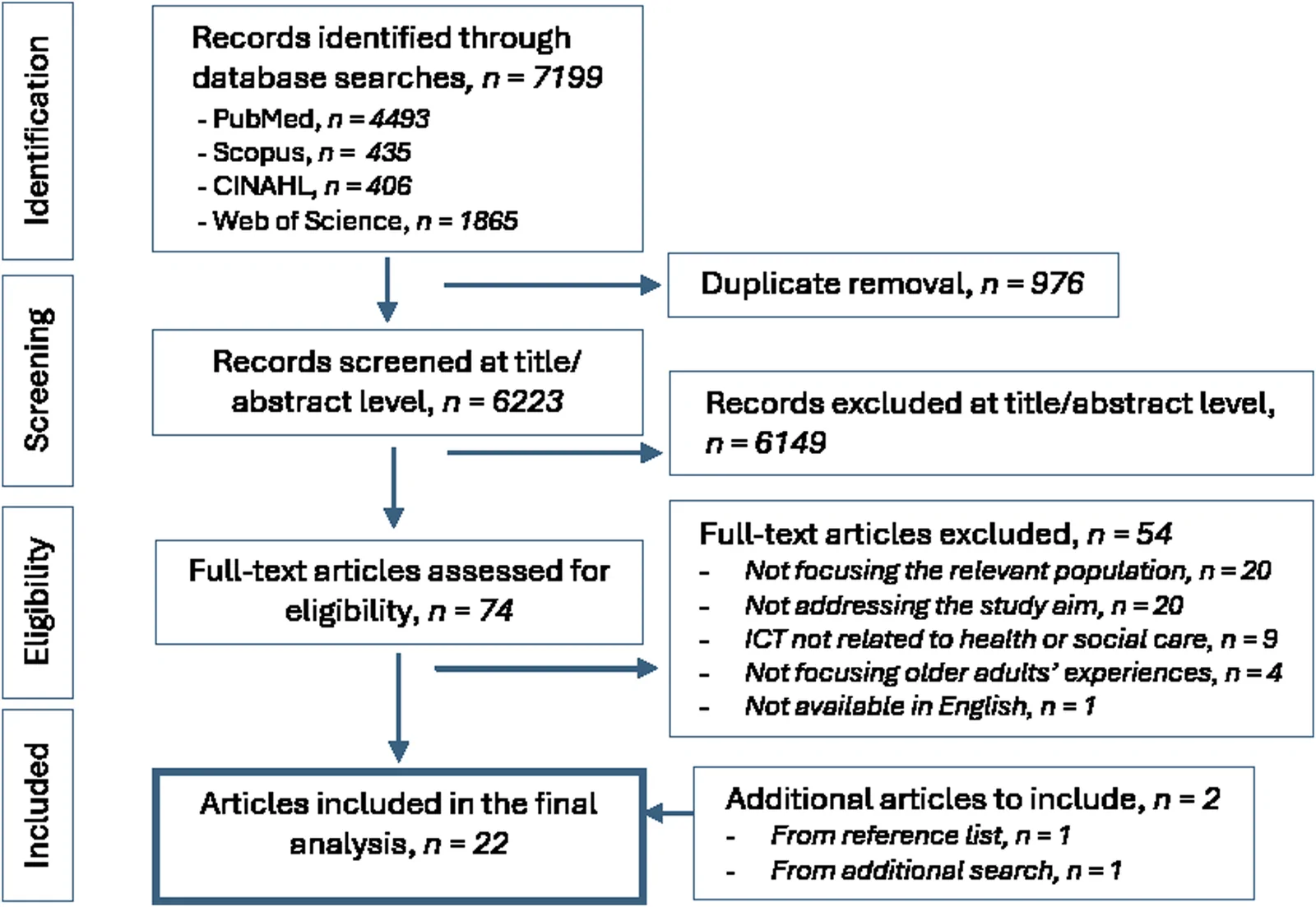
Flowchart of study selection.

All identified articles were exported to the Rayyan Screening Tool.^24^ In total, 7199 records were identified through database searches, and one additional record was identified through reference list screening. Duplicates were removed early in the screening process, resulting in 6223 records eligible for screening. The remaining records were independently screened at the title and abstract levels by the first and last authors, corresponding to the third step of Arksey and O'Malley’s framework,^17^ resulting in 74 articles for full-text assessment. Full texts were independently reviewed by the same authors, and any disagreements were resolved through discussion, with involvement of the second author, when necessary, until consensus was reached. All co-authors subsequently reviewed the included studies to confirm the accuracy of the selection. After reviewing the reference lists and conducting an additional search, two more articles were included, resulting in a final set of 22 articles in the analysis (Figure 1).

### Analysis

The fourth step of the Arksey and O'Malley framework^17^ consists of data charting, during which relevant information was systematically extracted and recorded from the 22 selected studies. A structured data charting form was developed using tables in Microsoft Word. Data charting was initially conducted by the first author, after which all co-authors reviewed the extracted data to ensure completeness and accuracy. Data were charted based on study characteristics and main findings. This systematic charting process enabled a comprehensive overview of the included studies and supported the subsequent analysis, which was conducted in accordance with the fifth step of the Arksey and O'Malley framework,^17^ involving summarizing and reporting the results. Guided by the review question, the data were analyzed and organized according to similarities in content by the first and last authors, resulting in a preliminary narrative summary describing frail older adults’ challenges in using telehealth services. Based on this summary, the first and last authors, together with the second author, drafted an initial version of the manuscript. The draft was subsequently reviewed by all co-authors, who also approved the final version. In accordance with Arksey and O’Malley’s framework^17^ and the PRISMA-ScR^18^ guidelines, the findings are presented as a narrative summary.

## Results

The results are presented in two sections: one presenting the characteristics of the articles included, and another describing the five domains of challenges experienced by frail older adults in using telehealth services.

### Study characteristics

The sample included in the review consisted of 22 articles^25–46^ with publication years ranging from 2016 to 2025, with the greatest number of studies being published in 2025 (*n* = 7). The studies included were conducted across three continents, with Europe being the most frequently represented (Table 1).

**Table 1.** Characteristics and charted data of included studies.

Author, Year, Title, Reference, Country	Aim	Study design, Context	Participants	Type of digital health services	Perceived challenges with digital health services
**Canet-Velez **et al., 2023^25^ Experience, facilitators, and barriers to the implementation of a multi-component programme in older people living in the community, +AGIL Barcelona: A qualitative study Front. Public Health 2023;1:1161883.https://doi.org/10.3389/fpubh.2023.1161883 Spain	- To explore the perspective of frail older adults and professionals about the barriers, facilitators, and improvement elements of the development of the +AGIL Barcelona program	Qualitative study; focus group- and individual interviews Community care Two focus groups and one individual interview were performed with frail older adults who had participated in at least half of the +AGIL Barcelona program.	The sample comprised 11 frail older adults (mean age 83 years; 91% female); the majority were vulnerable or mildly to moderately frail. Frailty-assessment: Clinical Frailty Scale	Evaluation of a multicomponent comprehensive geriatric assessment care program (+AGIL Barcelona), including the Vivifrail app, a publicly available exercise application tailored to baseline physical function	• Limited digital access and digital skills. • Lack of technical support. • Basic personal devices
**Chee **et al., 2024^26^ Age-related digital disparities, functional limitations, and social isolation: Unraveling the grey digital divide between baby boomers and the silent generation in senior living facilities Aging Ment. Health 2024; 28(4):621-632.https://doi.org/10.1080/13607863.2023.2233454 Malaysia	- To explore the lived experiences of older adults as they confront the challenges posed by age-related digital disparities inherent in the gray digital divide in senior living facilities	Qualitative study; individual interviews Phenomenological approach Senior living facilities	The sample comprised 28 older adults ≥60 years old, living in six senior living facilities in three urban locations. Participants had diverse functional levels, often requiring assistance with daily activities and multiple health conditions. No frailty assessment	Digital technology and services used in senior living facilities	• Limited access to devices and internet connectivity. • Language and financial barriers. • Low digital confidence and security concerns. • Limited digital skills. • Functional limitations affecting technology use
**Chen** et al., 2025^27^ Digital inclusion pathways and influencing factors among older adults in outpatient settings: A grounded theory study Clin. Interv. Aging 2025; 20:701–716.https://doi.org/10.2147/CIA.S518045 China	- To investigate the experiences, attitudes, and perceptions of older adults in outpatient care regarding smart healthcare service in order to develop an explanatory digital inclusion pathway and construct a comprehensive path model for older adults	Qualitative study; semi-structured individual interviews conducted in a hospital outpatient setting; separate interview guides for users and non-users of smart health services.	The sample comprised 27 older adults 60–85 years old who visited doctors at a smart hospital outpatient clinic. No frailty assessment	Smart healthcare services, such as digital appointment scheduling, online payment, medical report access, and self-service drug dispensing	• Physical and cognitive limitations. • Low digital health literacy and education. • Poor usability and complexity of smart services. • Financial and security concerns. • Lack of social and family support. • Technology anxiety, and low confidence
**Clancy** et al., 2023^28^ Benefits and barriers of technology for home function and mobility assessment: Perspectives of older patients with blood cancers, caregivers, and clinicians JCO Clin. Cancer Inform. 2023; 7: e2200171.https://doi.org/10.1200/CCI.22.00171 USA	- To qualitatively assess in focus groups of key stakeholders the benefits of and barriers to implementation of technologies for home function and mobility assessment in older adults with blood cancers	Three semi-structured qualitative focus groups were conducted; analyses included only data from the two groups comprising older patients.	The sample comprised eight older patients with either myelodysplastic syndrome or multiple myeloma and their caregivers (four patients and four or three caregivers in each focus group). Patients were classified as pre-frail.	Technology for home functional assessment included an intermittent wearable sensor and a passive monitoring device	• Reliance on support Concerns about data security and privacy. • Difficulty operating new technology. • Reliance on support
**Dequanter** et al., 2022^29^ Determinants of technology adoption and continued use among cognitively impaired older adults: A qualitative study BMC Geriatr. 2022; 22:376.https://doi.org/10.1186/s12877-022-03048-w Belgium	- To bridge gap in research by examining the factors underlying Technology use in community-dwelling older adults with mild cognitive impairments	Qualitative study; individual interviews with community-dwelling older adults with mild cognitive impairments.	16 community-dwelling older adults (mean age 76 years) with mild cognitive impairment or mild dementia; seven interviews included informal caregivers. No frailty assessment.	Participants were shown images of technological devices and asked to comment on the technologies and perceived facilitators and barriers to use	• Low digital literacy and self-efficacy. • Technical concerns (battery/charging) • Lack of internet access. • Limited perceived usefulness or relevance. • Physical limitations. • Financial constraints. • Trust, privacy, and Internet security concerns
**Ding** et al., 2025^30^ Experience of older adults using smart devices and mHealth apps in nursing homes in Chinese megacities: A descriptive qualitative study. Digit Health. 2025; 20(11): 20552076251353334.https://doi.org/10.1177/20552076251353334 China	- To explore the experiences of older adults in nursing homes located in megacities of China regarding the use of digital health tools	Qualitative study; semi-structured individual interviews conducted in nursing homes in Chinese megacities.	12 participants (aged 65–92 years) living in Chinese long-term care facilities. No frailty assessment	Mobile health (digital health tools)	• Unreliable or inaccurate technologies. • Low trust in digital health information. • Limited digital skills and confidence. • Technical support makes it easier to use
**Emmesjö** et al., 2025^31^ Older adults’ digital technology experiences: A qualitative study BMC Digit. Health 2025; 3:24.https://doi.org/10.1186/s44247-025-00163-7 Sweden	- To describe the experiences of using digital technology among older persons receiving health and social care at home	Qualitative semi-structured individual interviews with older adults from 12 municipalities.	17 older adults (aged 72–101 years) receiving daily municipal home care and municipal home healthcare services. No frailty assessment	Digital technology within municipality care	• Physical limitations. • Communication challenges. • Negative attitudes toward technology. • Financial concerns
**Goransson** et al., 2018^32^ Testing an app for reporting health concerns: Experiences from older people and home care nurses Int. J. Older. People Nurs. 2018; 13(2):e12181.https://doi.org/10.1111/opn.12181 Sweden	- To explore the experiences of using the Interactor app among older people with home-based healthcare and their nurses	Qualitative study; individual interviews with older adults only (healthcare provider focus group data excluded). Municipal care	17 older adults (aged 70–101 years) with multiple comorbidities (e.g., cardiovascular disease, diabetes, and stroke). No frailty assessment	The Interactor app enabled older adults to report health concerns via smartphone or tablet, with data transmitted securely to home care nurses	• Usability challenges (small fonts/buttons). • Initial frustration when learning to use devices and apps. • Fear of reduced in-person care.
**Gybel-Jensen** et al., 2024^33^ Patients’ experiences with digitalization in the health care system: Qualitative interview study J. Med. Internet Res. 2024; 26:e47278.https://doi.org/10.2196/47278 Denmark	- To examine digital practices and experiences with public digital health services and digital tools from the perspective of patients in the neurology field and to address the following research questions: 1) How do patients use digital services and digital tools? 2) How do they experience them?	Qualitative study; individual interviews in neurological hospital care (acute and transitional care).	31 interviews with patients hospitalized or previously hospitalized for neurological disease (e.g., stroke and dementia); mean age 71.8 years; 10 (32%) had physical, cognitive, or communicative impairments. No frailty assessment.	Public digital health services and digital tools	• Limited access and digital skills. • Challenges with security and login procedures. • Reliance on support for technical tasks. • Physical and cognitive limitations. • Fear of errors and fraud • Concerns about replacing human interaction
**Huang** et al., 2016^34^ Homebound patients’ perspectives on technology and telemedicine: A qualitative analysis Home Health Care Serv. Q. 2016; 35(3-4):172-181.https://doi.org/10.1080/01621424.2016.1264341 USA	- To investigate the attitudes of homebound patients to technology and telemedicine in order to identify and characterize impediments to telemedicine acceptance	Qualitative study; individual interviews with homebound patients, conducted at home or in healthcare facilities.	17 patients receiving visiting nurse services ≥1 month (aged 25–86 years; women 59%). Frailty not assessed but addressed in relation to homebound status.	Health technology and telemedicine	• Lack of devices and digital familiarity. • Low confidence in learning new technology. • Age- and function-related usability challenges. • Low perceived usefulness. • Preference for in-person care
**Kaihlanen** et al., 2022^35^ Towards digital health equity: A qualitative study of the challenges experienced by vulnerable groups in using digital health services in the COVID-19 era BMC Health Serv. Res. 2022; 22:188.https://doi.org/10.1186/s12913-022-07584-4 Finland	- To examine the challenges experienced by vulnerable groups in using digital health services during the COVID-19 pandemic	Qualitative study; individual interviews in Finnish public health services; recruitment via service organizations.	In total, 74 participants; findings limited to older adults (n = 19) and high users of health services (n = 17), mean age 75.4 years. No frailty assessment	Digital health services during the COVID-19 pandemic	• Limited digital skills. • Lack of devices and e-identification • Insufficient support and training. • Health and sensory impairments. • Fear and distrust of remote care. • Low awareness of digital services. • Preference for face-to-face care.
**Mahmoud** et al., 2024^36^ How can we improve comprehensive geriatric assessment for older people living with frailty in primary care and community settings? A qualitative study BMJ Open. 2024; 14:e081304.https://doi.org/10.1136/bmjopen-2023-081304 United Kingdom	- This qualitative sub-study aimed to understand perspectives, beliefs and experiences, of both older people and health professionals, to improve comprehensive geriatric assessment and explore factors that may impact on the delivery in community settings	Qualitative individual interviews via video, phone, or in person, in community care; only older adults’ perspectives were analyzed; recruitment from two epidemiological cohorts of community-dwelling older adults.	14 older adults (aged 75–90 years; 50% women) with sensory or mobility impairments and ≥1 long-term condition. Frailty was assessed using Edmonton Frailty Scale, Electronic Frailty Index, or Tilburg Frailty Indicator.	Experiences of technology use within comprehensive geriatric assessment and care	• Financial constraints limited access to technology. • Lack of devices and Internet connectivity. • Need for support. • Preference for in-person contact and non-digital formats
**Ong** et al., 2025^37^ A qualitative study on the impact and participation in the AGELESS multidomain intervention: Insights from older adults with cognitive frailty and their caregivers BMC Public Health. 2025; 25:7.https://doi.org/10.1186/s12889-024-20704-5 Malaysia	- To explore the insights of older adults with cognitive impairment and their caregivers regarding the impact and participation in the AGELESS multidomain intervention	Qualitative study using individual interviews with community-dwelling older adults; conducted as part of a randomized controlled trial evaluating a frailty-reversal intervention.	17 frail older adults with cognitive impairment (mean age 69 years; 58% women; Mini Mental Test score, 19–25). Frailty assessed using Fried criteria (≥1 criteria)	Study of the AGELESS 24-month multidomain intervention including expectations of remote participation, technology not clearly specified	• Limited digital literacy and competence. • Technical challenges (e.g., audio/video quality and unclear instructions). • Preference for face-to-face communication
**Portz** et al., 2020^38^ Perceptions of patient portal use for advance directive documentation among older adults with multiple chronic conditions J. Soc. Work End Life Palliat. Care. 2020; 16(3):238–249.https://doi.org/10.1080/15524256.2020.1771806 USA	- To 1) understand older adults’ use of an advanced care planning patient portal section and 2) obtain user design input on advance directive documentation features	Qualitative study using six semi-structured focus group interviews.	24 participants (mean age 78.4 years), long-term digital patient portal users with multiple chronic conditions (Charlson’s Comorbidity Index >2) No frailty assessment	Use of a patient portal for advance care planning and advance directive documentation	• Low awareness and understanding of technology involved. • Poor portal usability. • Security concerns. • Preference for non-digital storage. • Need for professional and technical support. • Difficulty verifying document access
**Rauzi** et al., 2024^39^ Older veteran experiences of using technology during a multicomponent telerehabilitation program: A convergent mixed methods study Cogent Gerontol. 2024; 3(1): 2340549.https://doi.org/10.1080/28324897.2024.2340549 USA	- To explore how older veterans engaged with technologies that were used during a multicomponent telerehabilitation program	Mixed-methods study; only qualitative data (semi-structured individual interviews) were included in this scoping review.	18 veterans (aged ≥60 years) with multiple medical conditions (≥3) and impaired physical function. No frailty assessment	Technology within the multicomponent telerehabilitation program	• Low digital self-efficacy. • Technical and connectivity issues. • Lack of integration between technologies. • Misunderstandings of technology functions. • Distrust in data accuracy
**Svärdh** et al., 2025^40^ “It is very convenient when it works - successes and challenges with welfare technology” - a qualitative study PLOS Digit. Health 2025:4(4): e0000844.https://doi.org/10.1371/journal.pdig.0000844 Sweden	- To explore how current welfare technologies challenge and/or facilitate support of older adults in their daily lives, as well as their healthcare staff in their work	Qualitative study; semi-structured individual interviews Municipal home care services	26 older adults (aged 65–99 years; 65% women) with multiple comorbidities receiving home care services from five Swedish municipalities. Analyses were limited to older adults (healthcare provider data excluded). No frailty assessment	Welfare technology: digital solutions supporting user independence and staff care processes	• Usability challenges • Maintenance and charging demands. • Distrust in reliability and security
**Terp** et al., 2021^41^ Older patients’ competence, preferences, and attitudes toward digital technology use: Explorative study JMIR Hum. Factors. 2021; 8(2): e27005.https://doi.org/10.2196/27005 Denmark	- To explore older patients’ readiness (i.e., competence, preferences, and attitudes) toward the use of information and communication technology, and to identify the factors that may act as barriers or facilitators for their engagement with health technology	Mixed-methods study; analyses included only data from semi-structured individual interviews; participants recruited from two internal medicine hospital units.	25 hospitalized older patients (median age 81 years; 52% women). No frailty assessment	Health-related information and communication technology	• Limited digital confidence. • Lack of interest or need. • Unfamiliarity with health information and communication technology. • Health-related limitations. • Linguistic challenges. • Low perceived usefulness
**Turjamaa** et al., 2023^42^ Older home care clients’ experiences of digitalisation: A qualitative study of experiences of the use of robot for medicines management Scand. J. Caring Sci. 2023; 37:561–570.https://doi.org/10.1111/scs.13141 Finland	- To describe older home care clients’ experiences of the implementation and use of a robot for medicines management at home	Qualitative study; semi-structured individual interviews in municipal home care services for older people in Finland.	38 older home care clients (aged 73–89 years), all living alone. No frailty assessment	A robot for medicine management at home	• Accessibility in the home environment. • Fear of learning failure. • Concerns about reduced professional support and loneliness. • Dependence on staff for medication management outside the home
**Wang** et al., 2016^43^ Mobile and wearable technology needs for aging in place: Perspectives from older adults and their caregivers and providers Nurs. Inform. 2016; 225:486–490.https://doi.org/10.3233/978-1-61499-658-3-486 USA	- To explore the perspectives of older adults and their caregivers or providers on the role of mobile and wearable technology tools for older adults aging in place	Qualitative study; individual interviews. The study was conducted at a retirement community with assisted living and independent living sections.	29 older adults (mean age 88 years) 17 in assisted living and 12 in independent living; approximately half in each setting moved to the facility due to age-related decline or medical illness. No frailty assessment	Wearable trackers and mobile devices	• High cost of health-tracking technology. • Perceived burden of learning how to use technology. • Concerns about durability and risk of device damage (e.g., exposure to water)
**Wang** et al., 2025^44^ Telehealth care for people with serious illnesses and preferred languages other than English JAMA Netw. Open. 2025; 8(9):e2529880.https://doi.org/10.1001/jamanetworkopen.2025.29880 USA	- To identify facilitators of and barriers to providing serious illness care via telehealth to people with preferred language other than English	Qualitative study; individual interviews. The study was conducted in an outpatient serious illness care setting delivered via telehealth at two palliative care clinics. For patients who were unable to participate themselves (e.g., due to dementia), their informal caregivers were included instead.	In total, 20 participants (10 patients and 10 informal caregivers) with a mean age of 64.7 (15.4) years. Analyses were limited to older adults. No frailty assessment	Telehealth visits conducted using Zoom videoconferencing	• Low digital literacy and difficulties using technology involved in virtual visits. • Language barriers, both English and digital language, complicated by hearing impairment. • Concerns about privacy and trust: feeling uncertain about who may be listening. • Dependence on relatives to facilitate telehealth visits
**Ward** et al., 2025^45^ Acceptability and feasibility of online delivery of chair-based yoga for older adults with multimorbidity: Lessons from a process evaluation of the gentle years yoga trial BMC Complement. Med. Ther. 2025; 25:107.https://doi.org/10.1186/s12906-025-04838-6 United Kingdom	- To undertake an in-depth qualitative exploration of participant and yoga teacher experiences and feasibility of the online delivery of the Gentle Years Yoga program	Qualitative study; observations and individual interviews; observations during a 12-week intervention with weekly yoga sessions. An initial individual Zoom session provided technical and safety support. In total, 30 interviews were conducted (one or two per participant).	18 participants, mean age 72 years Mean 4.9 comorbidities (range 2–8) No frailty assessment	Video conferencing; online delivery of a Yoga program for older adults with multiple long-term health conditions	• Inadequate equipment and space to perform yoga. • Technical and digital literacy challenges. • Access delays. • Financial constraints.
**Wildenbos** et al., 2019^46^ Mobile health for older adult patients: Using an aging barriers framework to classify usability problems Int. J. Med. Inform. 2019; 124:68–77.https://doi.org/10.1016/j.ijmedinf.2019.01.006 The Netherlands	- To assess usability problems that older patients encounter in two mobile health apps	Case-study approach with think-aloud tasks based on typical app use; data collected from video recordings and field notes.	13 participants from elderly homes; inclusion criteria: age >50 years, Dutch-speaking; app 2 required heart failure or chronic obstructive pulmonary disease. No frailty assessment	Two apps served as case models: 1) an app supporting older adults’ hospital appointment attendance; and 2) a self-monitoring app for chronically ill older adults	• Low digital confidence and motivation. • Cognitive limitations affecting navigation. • Perceptual difficulties (icons/text). • Fine-motor interaction challenges.

All studies had a qualitative approach using individual interviews (*n* = 19), focus group interviews (*n* = 3), or a combination of the two. One study used a case-study approach with video-recordings and field notes, and one study used observations from online yoga sessions in combination with individual interviews (Table 1).

Although in most studies, participants were described as having complex healthcare needs, including multimorbidity, disabilities, cognitive impairment, and dependencies, only four studies included a frailty assessment. The participants represented a range of contexts, such as community-dwelling settings, senior living facilities, hospital care, and municipal health services. In addition, some studies also included participants other than those who could be described as frail older adults. During the data extraction process, this was carefully considered, and only findings derived from data pertaining to the frail older participants were included in the results (Table 1).

In this study, the term telehealth is used as an umbrella concept encompassing all forms of digital healthcare services. The included studies addressed various telehealth modalities, most commonly exploring participants’ experiences of using information and communication technology (ICT) in general (Table 1).

### The main domains of experienced challenges

The analysis of the included studies identified five domains related to frail older adults’ experiences of challenges in telehealth services: 1) digital accessibility, 2) digital skills, 3) health status, 4) digital support, and 5) financial resources. A summary of the main findings is presented in Table 2.

**Table 2.** Summary of the main findings.

Domain	Summary of the main findings
Digital accessibility	Limited access to digital resources, internet connectivity, user-friendly technologies, and individual capabilities affected digital accessibility.^25,26,29,33–36,42,43,45^
Digital skills	Limited experience, confidence, learning opportunities, and operational skills affected the use of digital technologies and telehealth services.^25,26,30,32,33,35,37,39–41,44,45^
Health status	Physical, sensory, and cognitive impairments affected the ability to learn, use, and navigate digital technologies.^26,27,29,31,33–35,41,44,46^
Digital support	Informal and professional support were important for learning, navigating, and troubleshooting digital technologies.^25,27,28,30,35,36,38,44^
Financial issues	Cost of digital technologies, limited financial resources, and concerns about financial risks in digital healthcare services.^26,27,29,31,36,43,45^

### Digital accessibility

The analysis of the studies included showed accessibility-related challenges faced by frail older adults, particularly in relation to technology. Lack of access to digital devices or reliable internet connections was reported as a barrier to digital accessibility.^26,29,34–36,43,45^ In addition, low use of online digital technologies was reported among frail older adults. Exposure to technology was limited, and some older adults expressed reluctance to use it, alongside a need for more user-friendly solutions.^25^

Older adults reported that their digital devices contained few applications, limiting the opportunities available to them.^25^ The design and size of digital devices, such as robots for medication management, also posed challenges for older adults in the home environment, as they differed from such devices in the hospital setting.^42^ Furthermore, older adults struggled to participate in online chair-based yoga sessions due to limited access to adequate digital equipment and a suitable space for practice.^45^

In addition to these technology-related barriers, accessibility was also associated with functional limitations. For example, digital communication conducted in English was described as challenging.^26^ For older adults with mild cognitive impairment, accessibility was described as reduced when services require an Internet connection.^29^ Furthermore, cognitive and physical limitations were reported to affect how digital devices could be used in practice; for instance, when using smartphones, it may be difficult to see and feel small buttons, or to operate computers if illnesses affect hand function.^29,34^

### Digital skills

Frail older adults experience challenges related to digital skills when using digital devices. They have limited skills in using digital devices and applications, and describe infrequent engagement with digital technologies, as well as reluctance or resistance toward adopting such tools.^25^ Older adults who do not own digital devices report lacking the necessary skills to use them,^33,44^ and they also experience difficulties comprehending instructions on digital platforms.^37^

In addition, older adults experiencing frailty reported limited knowledge of telehealth services and limited experience of digital devices such as computers and smartphones during their working life.^26^ They also express concerns about the privacy of their personal health information when using telehealth services.^30,44^ For example, some described worries that sensitive health information could be compromised and described difficulties locating privacy settings.^30^

Several challenges are related to the practical use of digital devices. For example, small font sizes and limited button sizes on smartphones make them difficult to use, and it may take considerable time to learn how to log on.^32^ Digital safety alarms were mostly reported as easy to use, although difficulties activating the alarm button were also reported.^40^ Older adults also experience difficulties in understanding and interpreting digital language, following security procedures, and remembering passwords.^33,44^ Similarly, unfamiliarity with video-conference platforms can be challenging, particularly when handling passwords and virtual waiting rooms.^45^

Frail older adults with limited engagement in technology reported challenges learning and using digital technologies and described needing more time to learn.^39^ Some doubt their ability to learn or show little interest in engaging with new technology,^34^ while others report having previously acquired digital skills but later forgetting how to use them.^41^

### Health status

Among frail older adults, several health-related factors were reported as barriers to using and navigating digital devices.^33–35,41^ These included limitations in physical functioning,^26,27,29,31,34^ including hand deformities such as arthritis in the fingers and crooked fingers.^29,31,41^ Cognitive impairments,^27,33,46^ communicative inabilities,^33^ hearing loss,^27,31,44^ and impaired vision^27,29,31,41^ were also experienced as challenges among frail older adults.

Moreover, frail older adults reported that impaired physical functioning made it difficult to use telehealth services.^33–35^ Physical challenges included difficulties such as reading small text, using scrollbars, and interacting with small on-screen functions.^46^ In addition, older adults reported that poor health made it more difficult to learn and use telehealth services.^35^

Cognitive impairments represented an additional barrier. Older adults with health problems affecting memory and cognitive functioning describe difficulties using digital devices,^33^ such as difficulties remembering passwords, logging into systems, and keeping up with changes in digital services.^33^ These difficulties included locating information, understanding digital interactions, and attending to icons or messages.^46^ Participants also experienced difficulties understanding navigation structures and identifying important icons, buttons, and messages.^46^

In addition, deterioration in auditory function constrained older adults’ ability to use telehealth services^27^ and impaired vision posed further challenges. Older adults with sensory impairments reported difficulties using several digital device features, including small screens, such as those of tablets and smartphones.^36,41^

### Digital support

Older adults experiencing frailty describe seeking help from others when they experience a lack of knowledge about digital technologies,^27,28,30,35,44^ or to install, update, or acquire new devices.^33^ For example, older adults reported relying on family members, friends, and neighbors to set up new devices, transfer information, and manage practical technical issues.^33^ Furthermore, these older adults express a need for technical support that can provide guidance in their own homes,^25^ as well as assistance with navigating and troubleshooting digital devices.^38^ The absence of social and family support was reported as a barrier to using digital services independently,^27^ whereas some have support from family members they can rely on.^28^ Others receive support from caregivers but report the need for assistance from a broader network due to safety concerns.^36^

Older adults using telehealth services request better guidance from service providers.^35^ They also need professional assistance when they experience challenges completing advanced digital directives on their own.^38^

### Financial issues

Challenges for older adults in using telehealth services also include concerns about financial costs.^26,29,31,36,43^ This is associated with the cost of seeking health information digitally^43^ and the cost of digital technologies^31^ due to older adults’ limited financial resources.^36^ For example, some older adults reported being unable to afford technologies commonly used in everyday life,^36^ while others described the price of wearable health technologies as a barrier to their use.^43^ Older adults also expressed concerns about making costly mistakes when using digital payment systems and about reimbursement processes in digital healthcare services.^27^

## Discussion

The results of this study show that the challenges frail older adults experience in using telehealth services are not only technical but also arise from the interaction among frailty, limited digital access, low digital health literacy, inaccessible design, financial constraints, and insufficient human support.

Although the five domains were presented separately, the findings indicate considerable overlap between them. Health-related limitations, in particular, appeared to influence several other domains, including digital skills, engagement with digital services, and support needs. Physical, cognitive, visual, and hearing impairments were associated with difficulties in developing digital competencies, accessing digital services, and independently using telehealth technologies, resulting in a greater need for support. Importantly, assistance from family members, caregivers, and professionals could help compensate for challenges in other domains, whereas a lack of such assistance often reinforced them. Taken together, these findings support the broad understanding of frailty adopted in this study, encompassing both health deficits and functional impairments that influence independence and support needs.^20,22^ Accordingly, the barriers identified in this review should be understood as interconnected and mutually reinforcing rather than as isolated challenges.

Our findings indicate that frail older adults have limited experience in using digital technologies and telehealth services. Inadequate access to devices and Internet connectivity further restricts their opportunities for engagement and increases the risk of marginalization.^9^ This may contribute to reluctance to engage with digital technologies and concerns about online safety,^15^ while the difficulties are compounded by technical terminology, often in English, which many of the oldest adults did not learn in school.^47^

Older adults in better health may be more likely to use ICT and to seek information based on their own needs.^48^ The results of this scoping review indicate that health-related vulnerabilities, often associated with frailty, such as declining physical functioning, comorbidities, cognitive impairments, communication limitations, and sensory impairments (including of vision, touch, and hearing), can make the use of digital technologies particularly challenging.

Our study specifically focuses on this vulnerable group of older adults, but similar challenges have been described in the wider literature on aging and telehealth. Kahn et al.^49^ identified factors that hinder older adults from achieving adequate digital literacy, highlighting the role of health-related factors in learning and using digital technologies. Similarly, Hepburn et al.^50^ reported that low digital literacy, usability challenges, and concerns about privacy and trust influenced digital health adoption among older adults with chronic diseases. Although neither review^49,50^ specifically focuses on frailty, their findings are consistent with those of the present review and underscore the influence of health-related factors on digital inclusion. For digitalization in healthcare to be effective, the specific needs of older adults with functional limitations, need for assistance in daily life, and frailty must be considered.

Taken together, these challenges highlight the need for more accessible and user-friendly technologies. Simplified interfaces with clear, large text, clearly labeled buttons, and streamlined login procedures can help reduce cognitive load. Addressing these issues also requires the active involvement of older adults in the development and design of digital technologies, with adaptations introduced early through co-design processes.^51,52^ Accessibility features, such as adjustable text size, screen magnification, and compatibility with assistive devices, may therefore be implemented as standard rather than optional.

However, technological redesign alone is not sufficient. Building digital confidence among frail older adults requires sustained investment in education, training, and human support. Existing digital literacy programs are often not adapted to the specific needs, pace, and life context of older adults,^53^ leading to increased reliance on support from relatives. At the same time, research shows that informal caregivers may also perceive telehealth services and digital technology as stressful, highlighting the need for such support to be complemented by reliable information and guidance from healthcare professionals.^54^ Educational programs aimed at older adults and their relatives likely need to be designed around the lived realities of frail older adults, delivered in short, manageable sessions that account for fatigue, offered in familiar settings, and structured to allow repeated exposure to content, given that some participants may experience memory difficulties. Such digital skills programs could also be delivered online.^55^

Learning to use digital tools is an ongoing, nonlinear process that requires time and patience. Healthcare staff therefore need both the mandate and time to provide support without taking over tasks, as doing so may undermine learning and independence. Dedicated digital support services, provided at home or in community settings and potentially integrated into home care or delivered by digital coaches, may play a key role in addressing these needs.^56^

The strengths of this study include the use of an established methodological framework for scoping reviews^17^ and the transparency achieved through adherence to a pre-registered protocol. In addition, the review provides a comprehensive overview of the existing evidence, with a specific focus on challenges faced by frail older adults, who are often high users of healthcare services.

As interest in digital health and telehealth continues to grow, increasing attention has been directed towards understanding how older adults engage with these technologies. For example, recent reviews have examined challenges associated with digital health and telehealth use among older adults, including those with chronic conditions.^49,50^ However, less attention has been paid to frail older adults and those with health-related vulnerabilities. By focusing specifically on this population, the present review contributes a more detailed understanding of telehealth-related challenges among older adults with complex health and care needs.

Frailty, characterized by increased vulnerability and often associated with advanced age, functional limitations, and dependency, suggests that this group could particularly benefit from the accessibility offered by telehealth services. However, it was unexpected to identify so few studies in which participants had been screened for frailty using validated instruments. To capture the perspectives of this population, we therefore adopted a broader conceptualization of frailty and included studies in which participants exhibited characteristics strongly associated with frailty.^4^ Although these proxy characteristics aligned with domains included in established frailty screening instruments, the broader conceptualization inevitably introduced some heterogeneity in how frailty was identified across the included studies.

Nevertheless, some limitations should be considered. Relevant studies may have been missed due to the choice of databases, search strategies, and the restriction to peer-reviewed studies published in English, which was applied to ensure scientific quality. This may have led to the exclusion of potentially relevant research published in other languages or formats. In addition, variations in the terminology used to describe experiences with digital technologies may have influenced study identification, despite the use of a broad search strategy. Furthermore, differences in how frailty-related characteristics were operationalized across studies may have limited the comparability of the findings.

Taken together, these findings highlight an important equity consideration. While telehealth has the potential to improve access to care, without accessible design and structured support, it risks reinforcing exclusion among those with the greatest care needs. Future research may benefit from focusing on strategies to enhance inclusion and reduce inequalities in access to telehealth. This includes examining how healthcare professionals can best support frail older adults in using telehealth services, exploring the development of user-friendly technologies and applications, preferably through co-creation processes, and evaluating educational initiatives aimed at strengthening healthcare staff’s capacity to deliver equitable telehealth services.

## Conclusion

This scoping review shows that frail older adults experience multiple, interconnected challenges when using telehealth services, including limited digital access and skills, health-related limitations, insufficient support, and financial issues. These findings suggest that telehealth should not be implemented as a one-size-fits-all solution. Instead, services may be designed to better align with the needs, abilities, and life contexts of frail older adults. Accessible design, structured digital support, and affordable access to devices and Internet connectivity could be viewed as important components of inclusive telehealth provision. Involving frail older adults in the development and evaluation of telehealth services is also crucial to ensure that digital healthcare promotes participation and equity rather than reinforcing exclusion among those with the greatest care needs.

## Supplemental material

Supplemental material - Frail older adults’ challenges in using telehealth services: A scoping review Supplemental material for Frail older adults’ challenges in using telehealth services: A scoping review by Kristina Åhlund, Ann Svensson, Lilisbeth Perestelo-Perez, Ángel Acebes , Johannes Hobbelen, and Margareta Karlsson in Digital Health.

## Data Availability

Information on the review data is available by contacting the corresponding author.*
