# Supplementary material for: Frail older adults’ challenges in using telehealth services: A scoping review
Source: Digit Health. 2026 Aug 20;12:20552076261479799. doi: 10.1177/20552076261479799 (PMC13494289; doi:10.1177/20552076261479799)
Supplement: Supplementary file 1 [file sj-pdf-1-dhj-10.1177_20552076261479799.pdf]

Supplementary file

Search Strategies

Database: PubMed
Platform: National Library of Medicine
Date searched: August 14, 2025 (initial) and June 11, 2026 (updated search)
Time span: January 2015 to April 2026
Language limits: English
Document types: All document types in the initial search
Records Retrieved: n = 4493 (initial search), the updated search was limited to last year, clinical study, clinical trial, observational study, interview study, n= 217
Search: (Frail* OR multimorbidity OR comorbidity OR multi-disease* OR patient* complex needs OR senior* OR older adult* OR elderly) AND (Digital challenge* OR digital divide OR technology gap OR tech struggle* OR digital aversion OR digital barrier* OR digital competence OR digital tools accessibility OR digital exclusion) AND (digital* OR e-health OR m-health OR telehealth OR health informatics OR health information OR digital communication OR remote care OR telemedicine) AND (Experience* OR perception* OR perspective*)

Database: CINAHL with fulltexts
Platform: EBSCO host
Date searched: August 14, 2025 (initial search) and June 11, 2026 (updated search)
Time span: January 2014 to June 2026
Language limits: English
Document types: Academic journals in the initial search
Records Retrieved: n = 406 (initial search), the updated search was limited to last year, research articles and peer-reviewed, n = 121
Search: (Frail* OR multimorbidity OR comorbidity OR multi-disease* OR patient* complex needs OR senior* OR older adult* OR elderly) AND (Digital challenge* OR digital divide OR technology gap OR tech struggle* OR digital aversion OR digital barrier* OR digital competence OR digital tools accessibility OR digital exclusion) AND (digital* OR e-health OR m-health OR telehealth OR health informatics OR health information OR digital communication OR remote care OR telemedicine) AND (Experience* OR perception* OR perspective*)

Database: Web of Science Core Collection
Platform: Clarivate Analytics
Date searched: August 14, 2025
Time span: January 2015 to August 2025
Language limits: English
Document types: All document types
Records Retrieved: n = 1865
Search: (Frail* OR multimorbidity OR comorbidity OR multi-disease* OR patient* complex needs OR senior* OR older adult* OR elderly) AND (Digital challenge* OR digital divide OR technology gap OR tech struggle* OR digital aversion OR digital barrier* OR digital competence OR digital tools accessibility OR digital exclusion) AND (digital* OR e-health OR m-health OR telehealth OR health informatics OR health information OR digital communication OR remote care OR telemedicine) AND (Experience* OR perception* OR perspective*))

Supplementary file

Database: Scopus
Platform: Elsevier
Date searched: December 15, 2025
Time span: January 2015 to August 2025
Language limits: English
Document types: All document types
Records retrieved: n = 435
Search: (Frail* OR multimorbidity OR comorbidity OR multi-disease* OR patient* complex needs OR senior* OR older adult* OR elderly) AND (Digital challenge* OR digital divide OR technology gap OR tech struggle* OR digital aversion OR digital barrier* OR digital competence OR digital tools accessibility OR digital exclusion) AND (digital* OR e-health OR m-health OR telehealth OR health informatics OR health information OR digital communication OR remote care OR telemedicine) AND (Experience* OR perception* OR perspective*)
